# GRAM domain-containing protein 1A (GRAMD1A) promotes the expansion of hepatocellular carcinoma stem cell and hepatocellular carcinoma growth through STAT5

**DOI:** 10.1038/srep31963

**Published:** 2016-09-02

**Authors:** Binsheng Fu, Wei Meng, Hui Zhao, Bing Zhang, Hui Tang, Ying Zou, Jia Yao, Heping Li, Tong Zhang

**Affiliations:** 1 Department of Hepatic Surgery, Liver Transplant Center, the Third Affiliated Hospital of Sun Yat-sen University, Guangzhou 510630, P. R. China; 2 Liver Transplantation Center of Sun Yat-sen University, Guangzhou 510630, P. R. China; 3 Organ Transplantation Institute of Guangdong Province, Guangzhou 510630, P. R. China; 4 Department of Medical Imaging, the First Affiliated Hospital of Sun Yat-sen University, Guangzhou 510080, P. R. China; 5 Department of Medical Oncology, the First Affiliated Hospital of Sun Yat-sen University, Guangzhou 510080, P. R. China

## Abstract

Hepatocellular carcinoma (HCC) is the leading cause for cancer death worldwide, new prognostic factors and targets are critical for HCC treatment. Here, we found GRAMD1A was upregulated in HCC tissues, patients with high GRAMD1A levels had poor outcome, statistical analyses found GRAMD1A expression was positively correlated with pathologic differentiation and survival or mortality. It was an unfavorable prognostic factor for HCC patients. Functional analyses revealed GRAMD1A contributed to the self-renewal of HCC stem cells, resistance to chemotherapy and tumor growth of HCC determined by hepatosphere formation assay, side population (SP) analysis, TUNEL assay, soft agar growth ability assay and tumor growth model *in vivo*. Mechanism analyses found signal transducer and activator of transcription 5 (STAT5) was the target of GRAMD1A, GRAMD1A regulated the target genes of STAT5 and the transcriptional activity of STAT5. Inhibition of STAT5 in indicated HCC cells overexpressing GRAMD1A suppressed the effects of GRAMD1A on the self-renewal of HCC stem cell, resistance to chemotherapy and tumor growth, suggesting GRAMD1A promoted the self-renewal of HCC stem cells and the development of HCC by increasing STAT5 level. GRAMD1A might be a useful biomarker and target for HCC.

According to the data of GLOBOCAN 2012 provided by the IARC, about 782,5002 new HCC cases and 745,500 deaths occurred during 2014 worldwide. Almost 50% of new cases and deaths were Chinese, so the development of new prognostic factors and therapy targets is a big challenge for Chinese scientists^1^. Despite of the advances in diagnosis and treatment, the recurrence rate of patients after resection, chemotherapy and radiotherapy is still high due to high metastasis ability and treatment resistance. Our understanding of regulatory mechanisms of HCC initiation and progression is limited so far.

Recent years, a growing number evidences suggest cancer stem cells (CSCs) play a critical role in tumor relapse, CSCs are a small fraction of a tumor, and have the ability of initiation tumorigenecity, they can maintain the CSCs number or generate more CSCs by self-renewal, and differentiate into non-tumorigenic tumor cells to maintain tumor heterogeneity^2^,^3^. Chemotherapy and radiotherapy only kill non-CSCs, but CSCs still exist, so the tumors often relapse. Combining targeting CSCs drugs with chemotherapy or radiotherapy could kill all cancer cells^4^. HCC stem cells can be enriched using specific surface markers, like CD133^5^, CD90^6^, CD24^7^ and so on.

Many signaling pathways regulate the self-renewal of HCC stem cell and development of HCC, like annexin A3/ c-Jun N-terminal kinase (ANXA3/JNK) signaling promotes tumorigenesis, chemoresistance, metastasis, angiogenesis and self-renewal of HCC stem cells^8^. IL-6/Jak2/Stat3 pathway promotes HCC growth, chemoresistance and the self-renewal of HCC stem cells^9^,^10^,^11^. GRAMD1A first finds in human embryonic stem cell, and expresses in ectoderm, mesoderm and endoderm tissues and many tumor cells^12^, its function hasn’t been explored. Here, we studied the role of GRAMD1A in the prognosis, initiation and progression of HCC, we analyzed the correlation between GRAMD1A levels and many clinicopathological features, and examined whether GRAMD1A could function as a prognostic factor for HCC patients. Then we determined the role of GRAM1DA in the self-renewal of HCC stem cell and resistance to chemotherapy by overexpressing GRAMD1A, and the role of GRAMD1A in tumor growth by overexpressing or downregulating GRAMD1A. In the end, we studied its regulatory target, we found GRAMD1A was a prognostic factor for HCC, it promoted the self-renewal of HCC stem cell, resistance to chemotherapy and tumor growth through promoting STAT5, Inhibition of STAT5 in indicated cells with GRAMD1A overexpression inhibited the self-renewal of HCC stem cell, resistance to chemotherapy and tumor growth.

## Materials and Methods

### Ethics statement

All the clinical specimens were obtained with informed consent and approved by the First Affiliated Hospital of Sun Yat-sen University Ethics Committee, Informed consent was obtained from all patients involved in this study. Animal experiments were reviewed and approved by the Institutional Animal Care and Use Committee of Sun Yat-sen University. All the experiments were performed in accordance with the approved guidelines of the Institutional Research Ethics Committee of Sun Yat-Sen University.

### Tissue specimens and cell culture

A total of 96 human advanced HCC specimens were obtained from Department of Medical Oncology, the First Affiliated Hospital of Sun Yat-sen University. The detailed information was shown in Supplemental Table 1. Human HCC cells Huh7 and HepG2 were obtained from American Type Culture Collection (ATCC), and growth in DMEM medium supplanted with 10% FBS (GIBCO) and 1% penicillin-streptomycin (Hyclone).

### Vectors, oligonucleotides and transfection

The CDS of GRAMD1A was amplified form cDNA of human embryonic stem cell, and cloned into the pMSCV-Puro vector (indicated as GRAMD1A), the empty vector was used as negative control (indicated as Vector). small inference RNAs (siRNA) of GRAMD1A (indicated as siGRAMD1A) and their Scramble control were purchased from RiboBio Co. Ltd. The siRNAs were modified with 2′-OMe. The Vectors and oligonucleotides was transfected into Hun-7 and HepG2 using lipofectamine 2000 (Invitrogen).

### Quantitative real-time PCR

Total RNA was isolated using Trizol reagent (Life Technologies), cDNA was synthesized using PrimeScript RT Master Mix (Takara). Quantitative real-time PCR was performed using SYBR Green PCR Master Mix (Takara) on a ABI Prism 7500 Sequence Detection system (Applied Biosystems). The quantitative real-time PCR reactions were carried out in triplicate. GAPDH was used as an endogenous control to normalize the amount of mRNA in each sample. The relative expression was calculated with the 2^−ΔΔCt^ method. The primers for these genes were shown in Supplemental Table 2.

### Western blot

Tumor cells were homogenized with PIPA buffer (50 mM Tris (pH 8.0), 150 mM NaCl, 0.5% sodium deoxycholate, 0.1% SDS, 1% NP40 and 1 mM EDTA) supplemented with cocktail protease inhibitor (Roche). Western blot was performed according to the standard methods. The following antibodies were used: GRAMD1A (Sigma, HPA008852), caspase-3 (Cell Signaling Technology, #9662), poly(ADP-ribose) polymerase (PARP) (Cell Signaling Technology, #9542), BCL-XL (Cell Signaling Technology, #2762), CD133 (Miltenyi Biotec, 130-092-395), CD90 (Abcam, ab133350), STAT5 (Cell Signaling Technology, #9363), β-actin (Abcam, ab8226) and GAPDH (Abcam, ab8245).

### Luciferase reporter assay

The promoter sequence of STAT5 was amplified from genomic DNA prepared from 293T cells using PCR, and cloned into psi-CHECK2 vector (Promega). The vector was cotransfected into indicated cells with GRAMD1A overexpression vector or siRNAs of GRAMD1A using lipofectamine 2000 (Invitrogen). The luciferase activity was measured after 48 h of transfection using Dual Luciferase Assay (Promega) according to the manufacturer’s protocol.

### Soft agar growth ability assay

Soft agar growth ability assay was performed according to previously reported method^13^.

### Apoptosis assay

Cells were treated with various concentrations of Doxorubicin (0.5 uM and 1.0 uM, respectively) for 48 h. Following treatment, cells were harvested and stained with 0.1% Trypan blue, cell number was counted with a microscope. For TUNEL assay, slides were plated in six-well plates, then cells were seeded in the plates, and treated with 1.0 uM Doxorubicin for 48 h, after treatment, The TUMEL assay was performed using *In situ* Direct DNA Fragmentation Assay Kit (Abcam, ab66108) according to the instructions of manufacturer.

### Immunohistochemistry (IHC)

IHC was performed according to previous methods^14^,^15^. Anti-GRAMD1A antibody (Sigma, HPA008852) was used. The tissue sections were scored using two-blinded method. The proportion of tumor cells was scored as follows: Score 0, no positive cells; Score 1, 1–10% positive cells; Score 2, 11~50% positive cells; Score 3, 51–80% positive cells; Score 4, mane than 80% positive cells. The intensity of protein expression was shown as follows: 0 (no staining); 1 (weak staining, light yellow); 2 (moderate staining, yellowish brown) and 3 (strong staining, brown).

The staining index (SI) was calculated as the product of the staining intensity and the proportion of positive cell scores (scored as 0, 1, 2, 3, 4, 6, 8, 9 or 12). Cut-off values for GRAMD1A expression were chosen based on a measurement of heterogeneity using the log-rank test with respect to overall survival.

### Hepatosphere formation assay

200 Huh-7 or HepG2 cells were seeded in Ultra Low Attachment 6-well plates (Corning) and maintained with DMEM/F12 medium (Life Technologies) supplemented with 20 ng/ml human recombinant epidermal growth factor (Sigma), 10 ng/ml human recombinant basic fibroblast growth factor (bFGF, Millipore), 4 ug/ml insulin (Sigma), B27 (Life Technologies), 500 U/ml penicillin, 500 ug/ml streptomycin and 1% methylcellulose. Spheres were incubated in suspension for 2 weeks and counted under a microscope.

### Side population (SP) assay

Cells were resuspended at the density of 1 × 10^6^/ml in DMEM (Life Technologies), supplementing with 2% Fetal calf serum (FCS) (Life Technologies) and HEPES buffer (Life Technologies), and incubated with 5 ug/ml Hoechst 33342 dye in the presence or absence of Verapamil for 90 min at 37 °C with intermittent shaking. Then cells were washed using cold HBSS with 2% FCS and 10 mmol/L HEPES following centrifugation at 4 °C, and resuspended in cold HBSS with 2% FCS and 10 mmol/L HEPES. PI (propidium iodide) was added to gate viable cells. Cells were analyzed using a FACS Vantage-SE (BD).

### Animal studies

BALB/c-nu mice were purchased from the Experimental Animal Center of the Guangzhou University of Chinese Medicine. Xenograft tumors were established by subcutaneous injection of different number (1 × 10^5^, 1 × 10^4^ and 1 × 10^3^) Huh-7 cells into the flank of female BALB/C nude mice about 4-to-5 week old. Tumor sizes were measured every 6 days by calipers, tumor volumes were calculated according to the formula V = L × W^2^ × 0.5 (L: tumor length, W: tumor width). On day 31, animals were euthanized and tumors were excised. For orthotopic transplantation mouse model, 5 × 10^6^ Hub-7 cells with GRAMD1A knockdown or negative control were transplanted into the liver of mouse (n = 8) respectively, the mouse was fed for 40 days, the survival of mice was observed. The blood of mouse was extracted to investigate the concentration of ALT and AST.

### Statistical analysis

All statistical analyses were performed with SPSS 19.0 software (SPSS) or Excel (Microsoft). GRAMD1A expression data was downloaded from The Cancer Genome Atlas (TCGA) (https://gdc-portal.nci.nih.gov/projects/TCGA-LIHC). The Chi-square test and Fisher’s Exact test were performed to analyze the correlation between GRAMD1A levels and HCC clinical features. The Spearman correlation analysis was used to confirm the correlation between GRAMD1A levels and clinical features. Independent prognostic factors were examined by the Cox proportional hazards stepwise regression model. Survival curve was plotted by Kaplan-Meier survival analysis and compared by the log-rank test. Gene set enrichment analysis (GSEA) analysis was performed using online website (http://software.broadinstitute.org/gsea/index.jsp)^16^. Results were showed as the Mean ± SEM. A two-tailed paired student’s t test was used to assess the significant difference of two groups of data. A *p* value of less than 0.05 was considered statistical significance.

## Results

### GRAMD1A overexpression is positively associated with HCC progression

**Figure 1:**
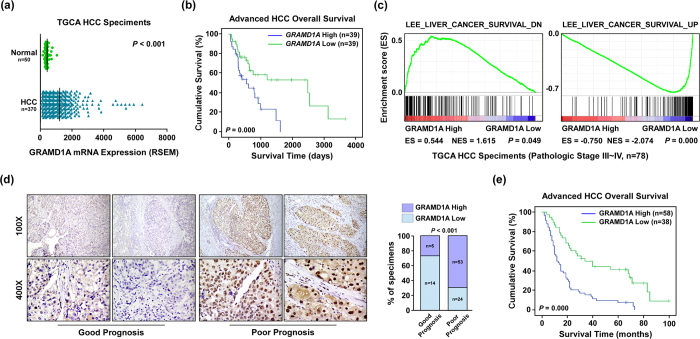
HCC patients with high GRAMD1A levels have poor outcome. **(a)** Analysis GRAMD1A mRNA levels in HCC tissues and normal liver tissues using TCGA data set. (**b)** Kaplan-Meier curves with log rank test based on the expression of GRAMD1A using TCGA data set. (**c)** Analysis of the correlation between GRAMD1A levels and survival state using GSEA, data was downloaded from TCGA data set (Pathologic stage III∽IV). (**d)** IHC assay of GRAMD1A levels in HCC tissues (top, 100X; bottom, 400X). Representative figure (*left*), statistical analysis of GRAMD1A levels in patients with good prognosis and poor prognosis (*right*). (**e**) Kaplan-Meier plots of overall survival based on the expression of GRAMD1A.

To determine the role of GRAMD1A in HCC progression, we used TCGA dataset to investigate GRAMD1A expression in HCC tissues and normal live tissues, and found GRAMD1A was significantly upregulated in HCC tissues (Fig. 1a). To examine the association between GRAMD1A expression and advanced HCC, we chose 78 patients with advanced HCC (Pathologic Stage III–IV) to analyze the association between GRAMD1A expression and survival time, the log-rank test suggested patients with high GRAMD1A levels had poor outcome (p = 0.000, Fig. 1b). GSEA was used to confirm this results, and found high GRAMD1A expression was positively correlated with low HCC survival and inversely correlated with high HCC survival (Fig. 1c). These results suggested GRAMD1A was associated with HCC progression.

To further demonstrate above results, a cohort of 96 advanced HCC tissues was used to study the role of GRAMD1A in clinical progression, IHC analysis suggested GRAMD1A expression was positive in 96.9% (93/96) of examined tissues, only 3% (3/96) was negative. GRAMD1A is upregulated in 60.4% (58/96) tissues, only 39.6% was low expression (Supplemental Table 3). We next analyzed the association between GRAMD1A levels and the clinicopathological features of HCC tissues, as shown in Table 1, high GRAMD1A was significantly correlated with pathological differentiation (p = 0.036 measured by Chi-square test, p = 0.043 measured by Fisher’s Exact test) and Survive or Mortality (p = 0.002, measured by both Chi-square test and Fisher’s Exact test) (Table 1). In contrast, GRAMD1A levels wasn’t associated with gender, age, clinical stage, TNM classification, cirrhosis or HbsAg. We used Spearman correlation analysis to confirm this result, and found high GRAMD1A levels was significantly correlated with pathologic differentiation (rs = 0.210, p = 0.040) and Survival or Mortality (rs = 0.329, p = 0.001), suggesting GRAMD1A was associated with HCC progression. IHC analysis also found GRAMD1A was overexpressed in HCC tissues of patients with poor prognosis compared to HCC tissues of patients with good prognosis (p < 0.001). The detail data was shown as follows: GRAMD1A was overexpressed in 26.3% (5/19) tissues of patients with good prognosis, but it’s overexpressed in 68.8% (53/77) tissues of patients with poor prognosis (Fig. 1d). The log-rank test also revealed patients with high GRAMD1A levels had poor outcome (p = 0.000, Fig. 1e), suggesting GRAMD1A might be a prognostic factor for HCC patient. Univariate Cox regression analyses revealed T classification (p = 0.007) and GRAMD1A level (p = 0.000) were poor prognostic factors for HCC patients, Multivariate Cox regression analyses further revealed they are independent prognostic factors for HCC patients (Table 2). Together, these findings revealed GRAMD1A is associated with HCC progression and is an independent prognostic factor for HCC patients.

### GRAMD1A promotes the self-renewal of HCC stem cells and resistance to chemotherapy

**Figure 2:**
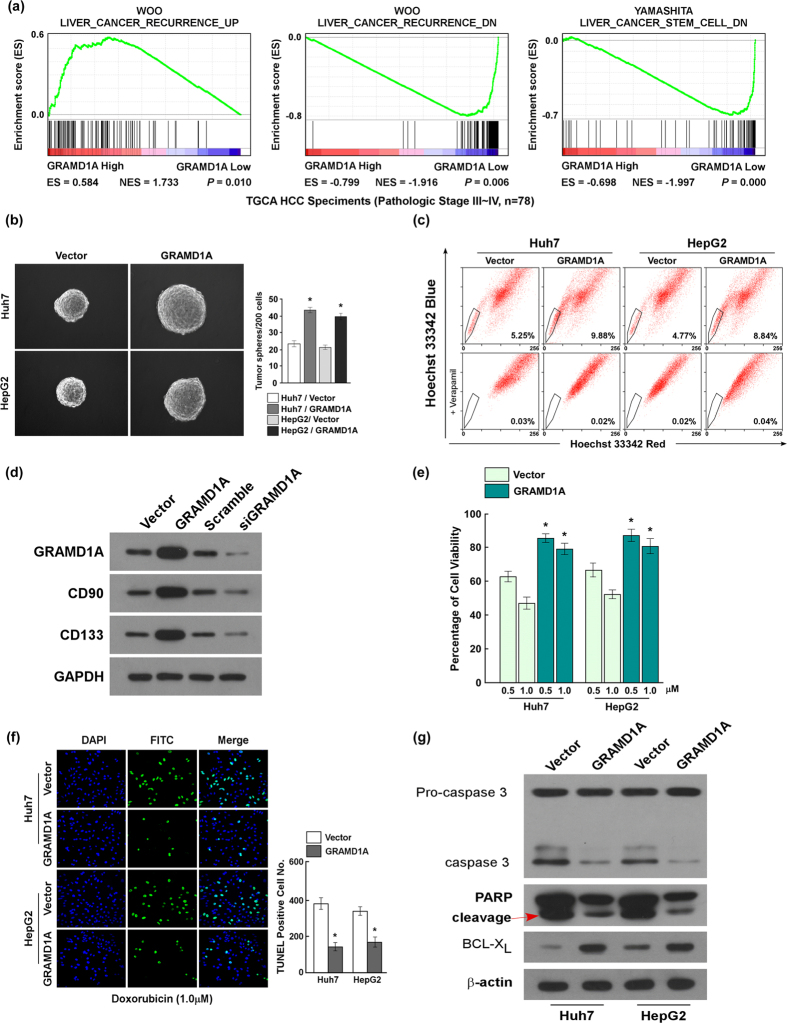
GRAMD1A promotes the self-renewal of HCC stem cell and the resistance to chemotherapy. (**a)** GSEA analysis of the correlation between GRAMD1A levels and tumor recurrence and HCC stem cells gene signature, data set was downloaded from TCGA data set. (**b)** Hepatosphere formation assay for the effect of GRAMD1A overexpression on self-renewal of HCC stem cells in Huh-7 and HepG2 cells. Representative micrographs (*left*); quantification of hepatosphere number (*right*). (**c)** SP analysis demonstrated the role of GRAMD1A in self-renewal of HCC stem cells by GRAMD1A overexpression. (**d)** Western blot assay of the expression of HCC stem cells markers CD130 and CD90 by overexpressing or downregulating GRAMD1A in HepG2. (**e)** Cell viability assay demonstrated the role of GRAMD1A in resistance to Doxorubicin by GRAMD1A overexpression. (**f)** TUNEL assay for the effect of GRAMD1A overexpression on resistance to Doxorubicin. Representative micrographs (*left*); quantification of TUNEL positive cell number (*right*). DAPI was used to stain nucleus. (**g)** Western blot assay for caspase 3 activity, PARP cleavage and the BCL-X_L_ levels after GRAMD1A overexpression in Huh-7 and HepG2. Data are expressed as mean ± SEM. *p < 0.05.

We used GSEA to analyze the association between GRAMD1A levels and tumor recurrence, and found patients with high GRAMD1A levels had high recurrence rate (Fig. 2a). Meanwhile, we found low GRAMD1A levels correlated with repressed self-renewal of HCC stem cell, suggesting GRAMD1A might regulate the tumor growth, drug resistance and the expansion of HCC stem cells. To determine GRAMD1A’s role in the initiation and progression of HCC, we overexpressed GRAMD1A in HCC cells Huh7 and HepG2. Hepatosphere formation assay suggested overexpression of GRAMD1A increased the number and volume of hepatosphere (Fig. 2b). SP assay found overexpression of GRAMD1A increased the number of SP population (Fig. 2c). The markers of HCC stem cells, CD90 and CD133, were used to demonstrate GRAMD1A regulates HCC stem cells, western blot assay found overexpression of GRAMD1A increased CD90 and CD133 levels, while knockdown of GRAMD1A decreased CD90 and CD133 levels (Fig. 2d). These results suggested GRAMD1A promoted the expansion of HCC stem cells. In order to investigate the effect of GRAMD1A on drug resistance, we use chemotherapy drug Doxorubicin (Sigma) to treat indicated HCC cells. Cell survival assay found after treatment with Doxorubicin (dissolved with DMSO, 0.5 uM and 1.0 uM, respectively), the number of survival cells with GRAMD1A was significantly more than negative control cells (Fig. 2e). Apoptosis assay was used to confirm this result, TUNEL assay found after treatment with Doxorubicin (1.0 uM), the number of TUNEL positive cells was significantly decreased compared to negative control (Fig. 2f). Cleavages of pro-caspase and PARP are the markers of apoptosis^17^,^18^, western blot assay found GRAMD1A overexpression inhibited the activation of caspase 3 and the cleavage of PARP, but the expression of an anti-apoptotic protein BCL-X_L_ was increased (Fig. 2g), suggesting GRAMD1A increased the drug resistance of HCC by inhibiting apoptosis. Together, these results suggested GRAMD1A promotes the expansion of HCC stem cells and resistance to chemotherapy.

### GRAMD1A promotes HCC growth

**Figure 3:**
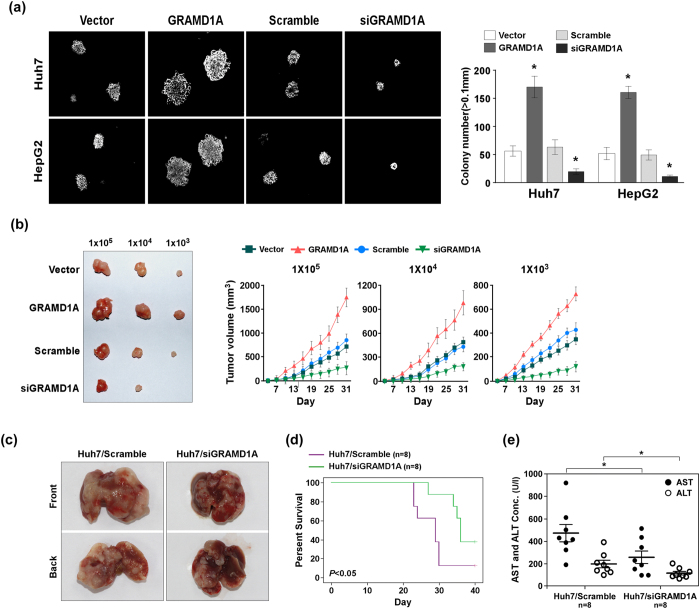
GRAMD1A promotes HCC growth. **(a)** Soft agar growth ability assay for the role of GRAMD1A in the anchorage-independent cell growth. Representative micrographs (*left*); quantification of colony number with diameter greater than 0.1 mm (*right*). (**b)** Xenograft tumors model for effect of GRAMD1A overexpression or knockdown on the growth of Huh-7 xenografts. Representative tumor images (*left*); the tumor volume (*right*). (**c)** Representative tumor images of orthotopic transplantation mouse model using Huh-7 with GRAMD1A knockdown and negative control. (**d)** Kaplan-Meier curves with log rank test for orthotopic transplantation mouse model. (**e)** the concentration of AST and ALT of orthotopic transplantation mouse model. Data are expressed as mean ± SEM. *p < 0.05.

We further determined the role of GRAMD1A in tumor growth. Soft agar growth ability assay suggested overexpression of GRAMD1A significantly promoted anchorage-independent growth, while knockdown of GRAMD1A significantly suppressed anchorage-independent growth (Fig. 3a). We injected subcutaneously different number of cells into the flank of nude mice, and found overexpression of GRAMD1A promoted tumor growth, the tumor volume was larger than negative control. While knockdown of GRAMD1A suppressed tumor growth, the tumor volume was smaller than Scramble control (Fig. 3b). We used orthotopic transplantation mouse model to conform this findings, knockdown of GRAMD1A inhibited HCC growth (Fig. 3c), survival cure analysis revealed the survival of mouse with GRAMD1A was significantly longer than Scramble group (Fig. 3d). We also analyzed the markers of liver injure, including ALT (Alanine aminotransferase) and AST (Aspartate aminotransferase), and found GRAMD1A knockdown significantly reduced the concentration of AST and ALT, confirming GRAMD1A promotes HCC growth (Fig. 3e).

### STAT5 is a downstream target of GRAMD1A

**Figure 4:**
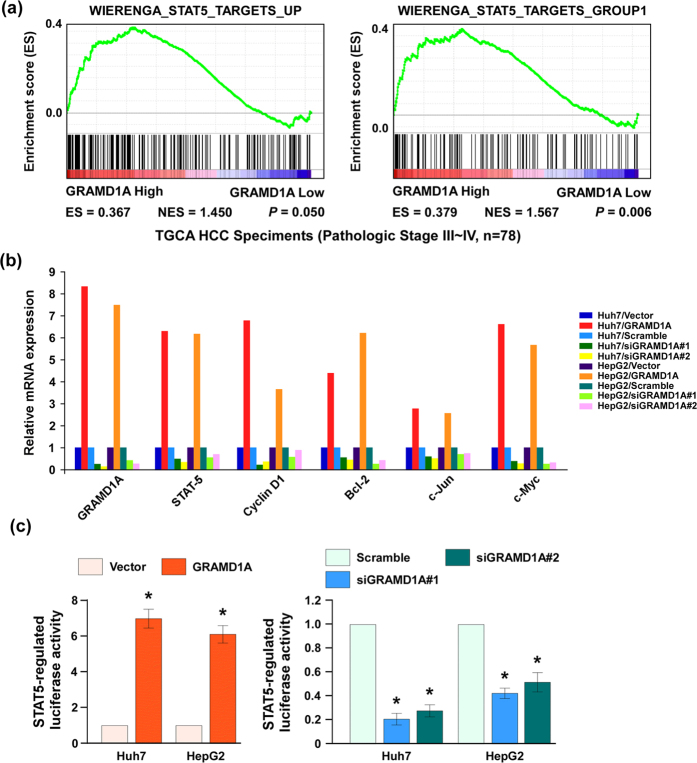
STAT5 is a downstream target of GRAMD1A. (**a)** Analysis of the correlation between GRAMD1A levels and STAT5 levels using GSEA. (**b)** Quantitative real-time PCR assay for the mRNA levels of STAT5, CyclinD1, Bcl-2, c-jun and c-Myc after GRAMD1A overexpression or knockdown. (**c)** Luciferase activity assay was used to determine whether GRAMD1A could regulate the transcriptional activity of STAT5. Data are expressed as mean ± SEM. *p < 0.05.

STATs play a key role in cell proliferation, differentiation, migration ad survival. STAT3 promotes HCC initiation and development, but the role of STAT5 in the initiation and development of human HCC has been reported rarely, we used TCGA data to analyze the relationship between GRAMD1A levels and the levels of STAT5 targeting genes by GSEA, and found a positive correlation between GRAMD1A levels and the levels of STAT5 targeting genes, suggesting GRAMD1A might regulate STAT5 (Fig. 4a). We also found overexpression of GRAMD1A increased STAT5 levels in both Huh-7 and HepG2 cells, while knockdown of GRAMD1A reduced STAT5 levels (Fig. 4b). Cyclin D1, Bcl-2, c-Jun and c-Myc are the target of STAT5^19^, GRAMD1A overexpression promoted these genes expression, while GRAMD1A knockdown inhibited these genes expression (Fig. 4b), suggesting STAT5 is a downstream of GRAMD1A. We cloned the promoter sequence of STAT5 into luciferase reporter vector, and transfected into indicated cells. Overexpression of GRAMD1A significantly increased luciferase activity, knockdown of GRAMD1A significantly decreased luciferase activity (Fig. 4c). These findings revealed GRAMD1A could regulate STAT5.

### GRAMD1A regulates the expansion of HCC stem cells, resistance to chemotherapy and tumor growth through regulating STAT5

**Figure 5:**
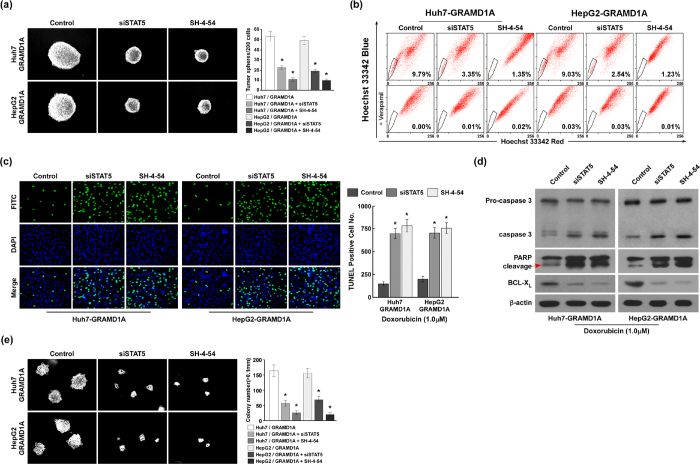
GRAMD1A promotes the self-renewal of HCC stem cells, resistance to chemotherapy and tumor growth. (**a)** Hepatosphere formation assay for the effect of STAT5 downregulation on self-renewal of HCC stem cells in Huh-7 and HepG2 cells with GRAMD1A overexpression. Representative micrographs (*left*); quantification of hepatosphere number (*right*). (**b)** SP analysis demonstrated the role of STAT5 downregulation in self-renewal of HCC stem cells in Huh7 and HepG2 with GRAMD1A overexpression. (**c)** TUNEL assay for the effect of STAT5 downregulation on resistance to Doxorubicin in Huh7 and HepG2 with overexpression. Representative micrographs (*left*); quantification of TUNEL positive cell number (*right*). DAPI was used to stain nucleus. (**d)** Western blot assay for caspase 3 activity, PARP cleavage and BCL-X_L_ levels after STAT5 downregulation in Huh-7 and HepG2 with GRAMD1A overexpression. (**e)** Soft agar growth ability assay for the role of STAT5 downregulation in the anchorage-independent cell growth of Huh7 and HepG2 with GRAMD1A overexpression. Representative micrographs (*left*); quantification of colony number with diameter greater than 0.1 mm (*right*). Data are expressed as mean ± SEM. *p < 0.05.

To investigate whether GRAMD1A regulates HCC initiation and development through regulating STAT5, we downregulated STAT5 in indicated HCC cells with GRAMD1A overexpression by siRNA for STAT5 and STAT inhibitor SH-4-54 (Selleckchem)^20^. Hepatosphere formation assay revealed downregulation of STAT5 significantly inhibited the number and volume of hepatosphere (Fig. 5a). SP analysis found downregulation of STAT5 reduced the percentage of the cell number of SP (Fig. 5b). These findings suggested GRAMD1A regulates the expansion of HCC stem cells through regulating STAT5. TUNEL assay revealed that TUNEL positive cells were significantly increased when STAT5 was downregulated in indicated cells with GRAMD1A overexpression (Fig. 5c). Western blot assay found downregulation of STAT5 promoted caspase 3 activation and PARP cleavage, and inhibited BCL-X_L_ levels (Fig. 5d). These findings suggested GRAMD1A regulated resistance to chemotherapy through regulating STAT5. Soft agar growth ability assay found downregulation of STAT5 significantly inhibited anchorage-independent growth (Fig. 5e), suggesting GRAMD1A regulates tumor growth through regulating STAT5.

## Discussion

GRAMD1A is a newly discovered gene, it’s function never been reported in the development of various diseases. In present study, we analyzed the role of GRAMD1A in HCC prognosis and progression, and found GRAMD1A was upregulated in HCC tissues, GRAMD1A expression was significantly positively correlated with pathologic differentiation and survival state, patients with high GRAMD1A had poor outcome, Further analysis revealed GRAMD1A was an independent prognostic factor for HCC patients.

We found GRAMD1A overexpression promoted the self-renewal of HCC stem cells, the resistance to chemotherapy and tumor growth. Here, we found GRAMD1A regulated the target genes of STAT5, such as CyclinD1, Bcl-2, c-jun and c-Myc. GRAMD1A also regulated the transcriptional activity of STAT5, overexpression of STAT5 promoted the STAT5 activity, while knockdown of STAT5 inhibited the STAT5 activity. This finding suggested GRAMD1A regulated STAT5 expression, but we couldn’t study whether GRAMD1A regulates the phosphorylation of STAT5 and the location of STAT5. The molecular mechanisms of GRAMD1A are still to be elucidated, the direct interaction proteins of GRAMD1A must be analyzed using co-immunoprecipitation coupled with tandem mass spectrometry.

Previous results have demonstrated that STAT5 which has two isoforms, STA5A and STAT5B, plays critical role in normal and malignant cells, it can be phosphorylated by Jak2, and causes it translocate to nuclear to regulate the transcription of STAT5′ target genes^21^,^22^. For example, STAT5 promotes the proliferation, survival and self-renewal of hematopoietic stem cell^23^,^24^. STAT5 increases the AR stability, promotes castration-resistant prostate cancer growth, tumor metastasis and the self-renewal of prostate cancer stem cells^25^,^26^,^27^.

The role of STAT5 in HCC progression is studied by several labs, but their results are contradictory. Lother Hennighausen, *et al*. find CCl4 induces liver tumorigenesis in STAT5 knockout mice by increasing TGF-β stability and STAT3 activity, the N-terminal of STAT5 interacts with TGF-β to decrease TGF-β levels, overexpression of N-terminal STAT5 inhibits HCC formation. TGF-β upregulation abrogates growth hormone-induced STAT5 activation. Instead, TGF-β upregulation promotes growth hormone-induced STAT3 activation^28^. Meanwhile, they find STAT5 is essential for growth hormone induced NOX4 expression, in turn induces ROS generation and proapototic proteins PUMA and BIM expression^29^. Other labs also find in growth hormone signaling hyperactivated mice, deletion STAT5 protects liver tumorigenesis^30^. Knockdown of STAT5 in HCC cell SMMC7721 inhibits cell proliferation and induce apoptosis^31^. These findings suggest STAT5 is a tumor suppressor for HCC. Other labs also find STAT5B level is positively correlated with HCC progression, overexpression of STAT5B promotes epithelial-mesenchymal transition (EMT) and invasion in HCC induced by HBV^32^. We found knockdown of STAT5 in Huh-7 and HepG2 with GRAMD1A overexpression suppressed the self-renewal of HCC stem cells, resistance to chemotherapy and tumor growth, suggesting STAT5 was an oncogene. The role of STAT5 in HCC might be context-dependent.

In summary, we provide evidences that GRAMD1A is a prognostic factor, it promotes the self-renewal of HCC stem cells, resistance to chemotherapy and tumor growth through regulating STAT5.

## Additional Information

**How to cite this article**: Fu, B. *et al*. GRAM domain-containing protein 1A (GRAMD1A) promotes the expansion of hepatocellular carcinoma stem cell and hepatocellular carcinoma growth through STAT5. *Sci. Rep.* **6**, 31963; doi: 10.1038/srep31963 (2016).

## Supplementary Material

Supplementary Information

## Figures and Tables

**Table 1:** Correlation between GRAMD1A expression and clinicopathologic characteristics of HCC.

Characteristics		GRAMD1A		Chi-square test *P*-value	Fisher’s Exact test *P*-value
		Low No. cases	High No. cases		
**Gender**	Male	30	46	1.000	1.000
	Female	8	12		
**Age (years)**	>45	24	44	0.251	0.251
	≤45	14	14		
**Clinical Stage**	IIIa	21	37	0.296	0.296
	IIIb	14	13		
	IIV	3	8		
**T classification**	T1	15	20	0.973	0.973
	T2	7	12		
	T3	9	15		
	T4	7	11		
**N classification**	N0	23	30	0.411	0.411
	N1	15	28		
**M classification**	No	35	50	0.518	0.518
	Yes	3	8		
**Pathologic Differentiation**	Well	2	0	0.036	0.043
	Moderate	27	34		
	poor	9	24		
**Cirrhosis**	Yes	21	26	0.404	0.404
	No	17	32		
**HBsAg**	Yes	28	40	0.654	0.654
	No	10	18		
**Survive or Mortality**	Yes	11	3	0.002	0.002
	No	27	55		

**Table 2:** Univariate and multivariate analyses of various prognostic parameters in patients with HCC Cox-regression analysis.

	Univariate analysis			Multivariate analysis		
	No. patients	*P*	Regression coefficient (SE)	*P*	Relative risk	95% confidence interval
T classification						
T1	35	0.007	0.273 (0.102)	0.005	1.333	1.090–1.630
T2	19					
T3	24					
T4	18					
Expression of GRAMD1A						
Low expression	38	0.000	1.041 (0.248)	0.000	2.901	1.776–4.739
High expression	58					
