# Supplementary material for: GRAM domain-containing protein 1A (GRAMD1A) promotes the expansion of hepatocellular carcinoma stem cell and hepatocellular carcinoma growth through STAT5
Source: Sci Rep. 2016 Sep 2;6:31963. doi: 10.1038/srep31963 (PMC5009375; doi:10.1038/srep31963)
Supplement: Supplementary Information [file srep31963-s1.pdf]

GRAMD1A promotes the expansion of hepatocellular carcinoma stem cell and hepatocellular carcinoma growth through STAT5

Binsheng Fu[1,2,3*], Wei Meng[1,2,3*], Hui Zhao[1,2,3*], Bing Zhang[4], Hui Tang[1,2,3], Ying Zou[5], Jia Yao[1,2,3], Heping Li[5#], Tong Zhang[1,2,3#]

[1] Department of Hepatic Surgery, Liver Transplant Center, the Third Affiliated Hospital of Sun Yat-sen University, Guangzhou 510630, P. R. China.

[2]Transplantation Research Institute of Sun Yat-sen University, Guangzhou 510630, P. R. China.

[3]Organ Transplantation Research Center of Guangdong Province，Guangzhou 510630, P. R. China.

[4]Department of Medical Imaging, the First Affiliated Hospital of Sun Yat-sen University. Guangzhou 510080, P. R. China.

[5]Department of Medical Oncology, the First Affiliated Hospital of Sun Yat-sen University. Guangzhou 510080, P. R. China.

[*]These authors contributed equally to this work

[#]Corresponding author: Tong Zhan，Department of Hepatic Surgery, Liver Transplant Center, Third Affiliated Hospital of Sun Yat-sen University, Guangzhou 510080, P. R. China. E-mail：zhjeff72@sina.com. Heping Li，Department of Medical Oncology, the First Affiliated Hospital of Sun Yat-sen University. Guangzhou 510080, P. R. China. E-mail: drliheping@163.com

Supplemental table 1 Clinicopathological characteristics of HCC patient samples

	Number of cases
**Gender**	
Male	76
Female	20
**Age(years)**	
> 45	68
≤ 45	28
**Clinical Stage**	
IIIa	58
IIIb	27
IV	11
**T classification**	
T1	35
T2	19
T3	24
T4	18
**N classification**	
N0	53
N1	43
**M classification**	
Yes	11
No	85
**Pathologic Differentiation**	
Well	2
Moderate	61
Poor	33
**Cirrhosis**	
Yes	47
No	49
**HBsAg**	
Yes	68
No	28
**Survive or Mortality**	
Survive	14
Mortality	82

Supplemental Table 2. The primers for genes

Gene name	Primer sequence (5' -> 3')
c-Myc	F: GTCAAGAGGCGAACACACAAC<br>R: TTGGACGGACAGGATGTATGC
c-Jun	F: TCCAAGTGCCGAAAAAGGAAG<br>R: CGAGTTCTGAGCTTTCAAGGT
Bcl-2	F: GGTGGGGTCATGTGTGTGG<br>R: CGGTTCAGGTACTCAGTCATCC
Cyclin D1	F: CAATGACCCCGCACGATTTC<br>R: CATGGAGGGCGGATTGGAA
STAT5	F: GAACACCCGCAATGATTACAGT<br>R: ACGGTCTGACCTCTTAATTCGT
GRAMD1A	F: TCAGTGCTACGGCTCAGAG<br>R: GGGCGATCACATCTCCCAC

F: Forward; R: Reverse

Supplemental table 3 The expression of GRAMD1A in HCC

Expression of GRAMD1A	
Negative	3(3.1%)
Positive	93 (96.9%)
Low expression	38 (39.6%)
High expression	58 (60.4%)
